# Glycerophospholipid Profiles of *Allomyrina dichotoma* Larvae at Different Instars Based on Lipidomics and Transcriptomics Suggest a Promising Lipid Source

**DOI:** 10.3390/insects16121220

**Published:** 2025-11-29

**Authors:** Kui Fang, Jia-Nan Liu, Cong-Xu Wang, Tao Wang, Yu Pan, Shang Wang, Jing-Hui Xi

**Affiliations:** 1 College of Plant Science, Jilin University, Changchun 130062, China; fangkui0118@126.com (K.F.); jiananl22@mails.jlu.edu.cn (J.-N.L.); cxw24r@163.com (C.-X.W.); ypan@cemps.ac.cn (Y.P.); 2 Kunming Customs Techenical Center, Kunming 650228, China; 3 Shulan Agro-Tech Extension Center, Shulan 132600, China; 15567302700@163.com

**Keywords:** glycerophospholipid, *Allomyrina dichotoma* larvae, lipidomics, glycerophospholipid synthesis, novel lipid resource

## Abstract

The farming of insects has emerged as a novel source of proteins and lipids. The larvae of *Allomyrina dichotoma*, an important source insect, contain large concentrations of lipids; however, the constituents of these lipids are unknown. We used lipidomics and transcriptomics to analyze the deposition of glycerophospholipids in *A. dichotoma* larvae at different instars. A total of eight subclasses of 833 glycerophospholipid molecules were identified in *A. dichotoma* larvae. The results showed the lipid with the highest levels in *A. dichotoma* larvae was phosphatidylethanolamine (PE) followed by phosphatidylcholine (PC), and phosphatidylglycerol (PG). Glycerophospholipids in *A. dichotoma* larvae at three instars were clearly differentiated. Large amounts of PE and PC were deposited in Stage 3 larvae, and cardiolipin (CL), phosphatidylinositol (PI), phosphatidylserine (PS), and phosphatidic acids (PAs) showed a similar pattern. However, PG and phosphatidylinositol diphosphate (PIP) decreased in Stage 3 larvae. Furthermore, the metabolic pathways and the key genes related to glycerophospholipid synthesis were identified based on transcriptomes for *A. dichotoma* larvae. These results revealed the composition of glycerophospholipids. We also explored the molecular mechanism for deposition of glycerophospholipids during the developmental stages of *A. dichotoma* larvae. Our results provide insights for using *A. dichotoma* larvae to produce lipids.

## Simple Summary

This study addresses the unknown composition of lipids in the larvae of *Allomyrina dichotoma*, a promising insect for sustainable protein and fat production. The research aimed to identify the classes of glycerophospholipids, as well as changes in them across different larval growth stages, using advanced chemical and genetic analyses. Our results identified 833 glycerophospholipid molecules. The most abundant types were phosphatidylethanolamine (PE) and phosphatidylcholine (PC), both of which significantly increased in the third larval stage. However, phosphatidylglycerol (PG) and phosphatidylinositol diphosphate (PIP) decreased in the same stage. The study also linked these changes to specific genes and metabolic pathways in the larvae. In conclusion, this work reveals the detailed lipid profile of these beetle larvae and uncovers the molecular mechanism behind lipid accumulation as they grow, providing a scientific basis for lipid utilization in farming insects.

## 1. Introduction

As hydrophobic or amphipathic small molecules, lipids play essential structural and functional roles in cellular membrane composition, in energy storage, and in intra- and intercellular signaling [1]. There are >1000 lipid species in a single cell, and 10,000–100,000 species in tissues or organisms [2]. These lipids are widely valorized (or, monetized) in various ways. In food sources, lipids enhance appearance, flavor, juiciness, tenderness, and nutritional value. For example, triacylglycerols are the primary lipids in edible fats and oils. Some lipids are a functional ingredient; for instance, phytosterols improve the lipoprotein profile for consumers [3]. However, certain lipids serve as precursors of harmful compounds. In addition, lipids like phospholipids are utilized in various roles, including emulsifier, anti-dusting agent, viscosity modifier, wetting agent, release agent, and separating agent [4]. Hence, the composition of lipids contributes to any assessment of the functional value of lipid products.

Currently, intensive agricultural land cannot satisfy the growing global demand for lipids and proteins [5]. The mass farming of insects to provide lipid and protein sources for humans or animals has been a focus of attention for various reasons [6,7]. The insect lipid fractions, including antioxidants and ω-3 fatty acids (FAs), have high nutritional value [8], and have even been used for medical purposes [9]. Historically, many species of insects of Hymenoptera, Orthoptera, and Coleoptera have been important natural food sources for humans [10]. The global market for edible insects has expanded in recent years, and is expected to grow further, reaching USD 7.96 billion by 2030 [4]. In order to make use of edible insect lipids, it is necessary to understand their chemical composition and nutritional quality.

Lipidomes of an increasing number of insects have been well documented [11,12]. For example, in [13], lipidomes of some insects, such as *Aedes aegypti*, were analyzed to understand lipid metabolism and physiological functions. In addition, in [4], the lipid profiles of some edible insects, such as cricket species, were determined to understand their nutritional value. However, there remain many insects with edible potential which are still to be profiled.

The larvae of *Allomyrina dichotoma* could be used for the sustainable production of lipids and proteins. In Asia, they have been eaten for a long time, and they are listed in the Korean Food Standards Codex as novel food resource due to their nutritional and health advantages [14]. As edible insects, larvae of *A. dichotoma* have many positive properties; moreover, they produce bioactive compounds that could beneficial for both human and animal health [15]. In traditional medicine, this beetle is applied for its anti-hepatofibrotic, anti-neoplastic, anti-obesity, and anti-diabetic effects [9,16]. In [17], an extract from *A. dichotoma* larva was shown to lower liver insulin resistance, reduce the harmful effects of free fatty acids, and suppress liver fat production in diabetic mice. The dry biomass of *A. dichotoma* larvae consists of approximately 38.17% proteins and 32.72% lipids [18]. Additionally, *A. dichotoma* larvae at different developmental stages might synthesize different lipid species in different amounts. To maintain quality consistency and maximize the nutritional value of *A. dichotoma*, it is important and timely to obtain a more comprehensive lipidomic profile of larvae at different developmental stages.

Glycerophospholipids, the most abundant lipids, are composed of a polar head group, a glycerol backbone, and as many as two fatty acyl chains. One of the free OH groups on the phosphate moiety is esterified with another component like serine, choline, ethanolamine, inositol, or glycerol to generate glycerophospholipid phosphatidylserine (PS), phosphatidylcholine (PC), phosphatidylethanolamine (PE), phosphatidylinositol (PI), phosphatidylinositol diphosphate (PIP), or phosphatidylglycerol (PG) [19]. Another type of glycerophospholipid is cardiolipin (CL), which is formed by the combination of C1 and C3 of glycerol with two molecules of phosphatidic acid. Glycerophospholipids contribute significantly to cellular metabolism, signal transmission, and membrane transport [20,21]. Glycerophospholipids are typical lipids containing health-critical ω3 polyunsaturated fatty acyl chains; they are not only important for human health and nutrition, but also affect food sensory traits [8]. Therefore, it is worthwhile to determine glycerophospholipid profiles in insects.

Lipidomics has been used widely to describe the characteristics of lipid molecules in cells, tissues, and organisms, as part of a comprehensive body of research in the fields of metabolic diseases, nutritional health, and food science [22]. With respect to food quality and safety, lipidomics has been used for analysis of the composition and quality of lipids. In our study, we combined lipidomic and transcriptome analysis to explore the synthesis and accumulation of glycerophospholipid profiles of *A. dichotoma* larvae at different developmental stages. Studying the glycerophospholipid profiles of *A. dichotoma* is valuable for evaluating potential food and nutrition prospects.

## 2. Method

### 2.1. Rearing and Sampling of A. Dichotoma

The larvae of *A. dichotoma* were obtained from a laboratory-maintained strain. The beetles were reared with sawdust in a rearing chamber at 25 °C, 60% RH, on a 12-hL:12-hD photoperiod. The insects were sexed to ensure a balanced representation of gender. There is a distinct, darkly pigmented transverse groove which appears as V-shaped or linear on the ventral side of the anal segment of the male; however, the ventral anal segment of the female is flat and smooth, without a groove. Twenty insects (10 male larvae and 10 female larvae) were collected for each replicate, and there were a total of 4 biological replicates. After collecting, all insects were immersed promptly in liquid nitrogen followed by storage at −80 °C until further processing.

### 2.2. Lipid Extraction from A. dichotoma Larvae

Lipids were extracted via the methyl-tert-butyl-ether (MTBE) method. The mass of samples for lipid extraction is around 30–33 mg per pool. In brief, 20 μL of internal lipid standards (Splash Lipidomix, Merck, Darmstadt, Germany) was added to the samples, which were then homogenized at 4 °C using 200 µL of water and 240 µL of pre-chilled methanol. Next, 800 µL of MTBE was introduced to the samples, and the mixture was vortexed for 30 s. After that, samples were subjected to ultrasonication at 4 °C for 20 min and incubation for 30 min at 24 °C to facilitate phase separation. Then, the organic and aqueous phases were separated by centrifuging the sample at 14,000× *g* for 15 min at 10 °C. The top layer of organic solvent, which contained the extracted lipids, was collected and dried by evaporating using a nitrogen gas stream. Before the subsequent analysis, lipid extracts were re-dissolved in 200 μL of isopropanol/acetonitrile (9:1, *v*/*v*) and centrifuged at 14,000× *g* for 15 min to remove insoluble particulates. Finally, 3 µL of the supernatant was injected for LC-MS/MS analysis.

### 2.3. Untargeted Lipidomics for Lipid Analysis

Lipid extracts were analyzed using LC-MS/MS. In brief, lipid extracts were separated using a CSH C18 Column (1.7 µm, 2.1 mm × 100 mm, Waters, Milford, MA, USA). The mobile phase A consisted of acetonitrile and water in a 6:4 volume ratio, containing 0.1% formic acid and 0.1 mM ammonium formate; the mobile phase B was a mixture of acetonitrile and isopropanol in a 1:9 volume ratio, also with 0.1% formic acid and 0.1 mM ammonium formate, flowing at 300 μL/min. The gradient started with 30% of mobile phase B and was held for 2 min. Then, the mobile phase was increased linearly to 100% of mobile phase B in 23 min. Finally, the gradient was returned to 5% mobile phase B for 10 min. Mass spectra detection was performed using Q-Exactive Plus (Thermo Scientific, Bremen, Germany) in positive and negative ion modes. For the ESI parameters, the source temperature was 300 °C, the spray voltage was 3.0 KV, the capillary temperature was 350 °C, the S-Lens RF Level was 50%, and the MS1 scan ranges were set from 200 to 1800.

Equal amounts of samples from each group were mixed to form the quality control (QC) samples. Data quality assessment was performed by comparison of base peak chromatograms (BPCs) and by use of a correlation plot, multivariate control chart, relative standard deviation (RSD) for the QC sample, and principal component analysis. Hotelling’s T2 test was used for all samples. This confirmed the robustness of the instrumental system and enabled a high degree of experimental repeatability, thereby ensuring high-quality data for subsequent analyses (Supplementary Figures S1–S6). Extraction blanks were employed to ensure that no contamination was introduced throughout the sample preparation process.

### 2.4. Identification and Quantification of Glycerophospholipid Species

The identification of lipid species was performed at the MS1 level using Lipid Search 4.0 software, followed by matching MS/MS fragments. The annotation level did not allow distinguishing of isomers because isomeric components were differentiated only on the basis of retention time. Detection was carried out in both positive and negative ion modes. The mass parameters were set as follows: m-score threshold was set to 2.0, precursor tolerance was set to 5, product tolerance was set to 0.1, and quantification *m*/*z* tolerance was set from −0.01 to +0.01. The quantification of lipid species was performed using a semi-quantitative method normalized by internal standards (Supplementary Table S1), and lipid content was determined based on the wet mass of whole larvae.

### 2.5. RNA Extraction and Transcriptomics Analysis

According to the manufacturer’s instructions, the isolation of total RNA from samples was performed using Trizol reagent (Invitrogen, San Diego, CA, USA). The RNA was then purified with RNase-free DNase I (TaKaRa, Kyoto, Japan). The assessment of RNA sample purity, concentration, and integrity was conducted using 1% agarose gel and a Nanodrop spectrophotometer (Nanodrop Technologies, Wilmington, DE, USA). For RNA sample preparation, 1 μg of RNA per sample was used. Sequencing libraries were constructed with the NEBNext^®^ Ultra™ RNA Library Prep Kit, (NEB, Ipswich, MA, USA), and their quality was checked on an Agilent Bioanalyzer 2100 system (Agilent, Santa Clara, CA, USA). The sequencing of libraries were performed using an Illumina Hiseq 2000 platform, and paired-end reads were generated. Gene function was annotated based on databases that included NR (NCBI non-redundant protein sequences), Pfam (Protein family), KOG/COG/eggNOG (Clusters of Orthologous Groups of proteins), Swiss-Prot (A manually annotated and reviewed protein sequence database), KEGG (Kyoto Encyclopedia of Genes and Genomes), and GO (Gene Ontology). The levels of gene expression were quantified using total fragments per kilobase per million reads (FPKM).

### 2.6. Statistical Analysis

Significant differences were evaluated through one-way analysis of variance (ANOVA) with post hoc Tukey’s test and FDR correction using Graphpad Prism 10 software. Principal component analysis (PCA) was carried out in R studio (https://www.r-studio.com/) with a ‘ggplot 2’ package, and significant differences between the treatments in PCA plots were assessed using Adonis analysis with Bray–Curtis dissimilarity, while homogeneity of dispersions was checked using betadisper.

## 3. Results

### 3.1. Overview of Glycerophospholipids in A. dichotoma Larvae

Samples of *A. dichotoma* larvae contained a total of 833 glycerophospholipid species. Glycerophospholipids were classified into eight categories, including CL, PA, PC, PE, PG, PI, PIP, and PS (Table 1). The quantity of glycerophospholipid molecules across various subclasses varied greatly. In particular, PC had the largest count with 328 of glycerophospholipid molecules, at 33.03% of the total amount. PE had the second-highest number (223) of molecules, at 47.58% of total glycerophospholipids. PG contained 84 different types of molecules, with 9.77% of the total amount. Each of the other glycerophospholipid classes (i.e., 72 CLs, 57 PIs, 34 PSs, 21 PIPs, and 14 PAs) accounted for < 3% of the total.

### 3.2. Dynamic Changes in Glycerophospholipid Content of A. dichotoma During Larval Development

**Figure 1:**
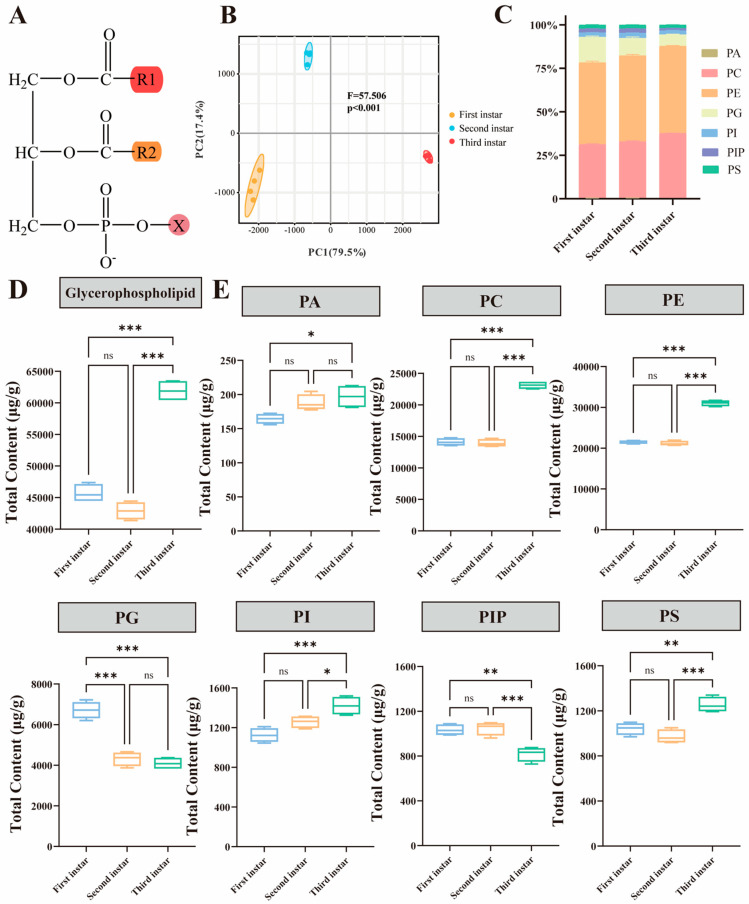
(**A**) Representative structure of glycerophospholipids. (**B**) Principal component analysis (PCA) plot of glycerophospholipids. (**C**) Relative composition of glycerophospholipid classes. (**D**) Total content levels of glycerophospholipid in *Allomyrina dichotoma* larval stages. One-way ANOVA was used for statistical analysis (*p* < 0.05 ‘*’; *p* < 0.001 ‘***’; ‘ns’ indicated no significant difference; *n* = 4). (**E**) Boxplot of total phosphatidic acids (PAs), phosphatidylcholine (PC), phosphatidylethanolamine (PE), phosphatidylglycerol (PG), phosphatidylinositol (PI), phosphatidylinositol diphosphate (PIP), and phosphatidylserine (PS) in *A. dichotoma* larval stages. One-way ANOVA was used for statistical analysis (*p* < 0.05 ‘*’; *p* < 0.01 ‘**’; *p* < 0.001 ‘***’; ‘ns’ indicated no significant difference; *n* = 4).

Glycerophospholipid content did not significantly change in the first- and second-instar larvae, whereas it significantly increased in the third-instar larvae (Figure 1D). PCA analysis of the glycerophospholipids showed significant separation among different instars (F = 57.51, R^2^ = 0.927, *p* = 0.002; *n* = 4) (Figure 1B). It is necessary to analyze the dynamic changes in each glycerophospholipid category during different larval stages (Figure 1C,E). PE and PC were the most abundant glycerophospholipids in larvae of all instars, followed by PG, with PA being the least abundant. The levels of PC and PE did not differ significantly between the first and second instar stages, but there was a significant increase in the levels of PC and PE, which reached 23,118.33 ± 602.74 ug/g and 310.25 ± 738.07 ug/g, respectively, for the third-instar larvae. Similarly, content levels of PI and PS increased significantly at the third instar stage, compared with the first and second instars. Compared to first-instar larvae, third-instar larvae had much higher PA content. Content levels of PG and PIP decreased significantly with increasing instar stages.

### 3.3. Changes in Cardiolipin (CL) in A. dichotoma Larvae During Different Developmental Stages

**Figure 2:**
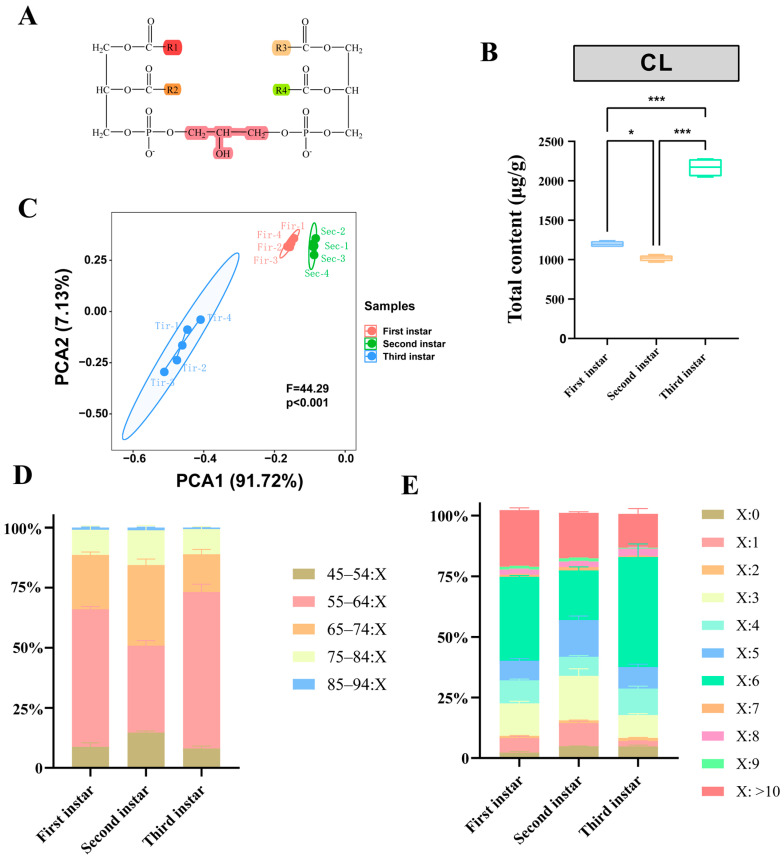
(**A**) Representative structure of cardiolipins. (**B**) Total content levels of cardiolipins in *Allomyrina dichotoma* larval stages. One-way ANOVA was used for statistical analysis (*p* < 0.05 ‘*’; *p* < 0.001 ‘***’; *n* = 4). (**C**) Plot of principal component analysis (PCA) of cardiolipins. Relative composition of cardiolipin species grouped by (**D**) the same degree of unsaturation (X:0–X:10) and by (**E**) the same acyl chain composition (45:X–94:X) of the constituent fatty acids.

PCA analysis of the CLs showed clear differences among different instars (F = 44.29, R^2^ = 0.908, *p* = 0.001; *n* = 4) (Figure 2C). The content of CLs in first-instar larvae was 1198.09 μg/g; this deceased markedly to 1018.48 μg/g in second-instar larvae, and increased significantly to 2168.07 μg/g in third-instar larvae (F = 257.7, *p* < 0.0001; *n* = 4) (Figure 2B).

The chain length of a lipid molecule is determined by the total number of carbon atoms in its fatty acid chains. This study categorized 72 CLs into five groups according to lipid chain lengths ranging from 45 to 94 (Figure 2D). CLs with chain lengths that ranged from 55–64 had the highest contents, with average values of 685.10, 367.59, and 1407.08 μg/g for first-, second-, and third-instar larvae, respectively.

The differences in cardiolipin content with different degrees of unsaturation among the three instars were compared further (Figure 2E). Most cardiolipin species had double bonds in each instar; CL species with six unsaturation bonds had the largest number of CLs, followed by CL species with three, five, and four unsaturation bonds. Except for species with zero, four, six, and nine unsaturation bonds, the CLs extracted from instar larvae contained lower levels of unsaturation CLs compared with first- and second-instar larvae. At chain unsaturation degrees of four and six, CLs in third-instar larvae were significantly greater than in second-instar larvae (*p* < 0.05).

### 3.4. Analysis of the Biosynthesis Genes Involved in Metabolic Pathways of Glycerophospholipids

**Figure 3:**
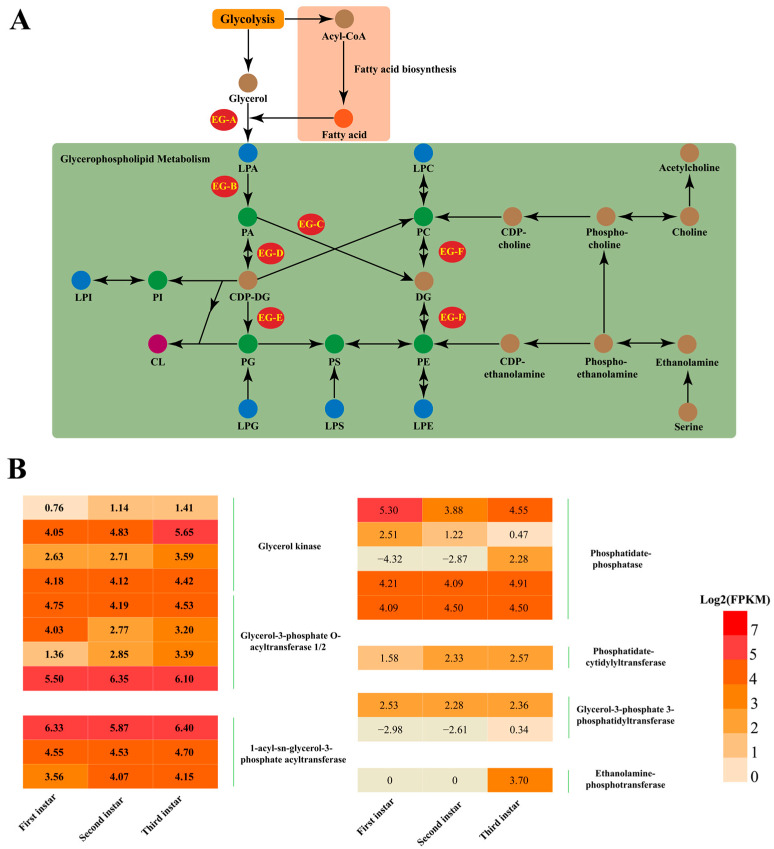
(**A**) Metabolic pathways involved in fatty acid biosynthesis and glycerophospholipid analysis. (**B**) Heatmaps of gene response to the glycerophospholipid metabolic pathway. Intensities of single genes are displayed using a color scale that ranges from red (higher values) to orange (lower values), as shown in the legend. Log2 (FPKM value) is shown in the heatmap. EG-A: glycerol kinase, glycerol-3-phosphate O-acyltransferase 1/2; EG-B: glycerol-3-phosphate O-acyltransferase 1/2; EG-C: phosphatidate-phosphatase; EG-D: phosphatidate-cytidylyltransferase; EG-E: glycerol-3-phosphate 3-phosphatidyltransferase; EG-F: ethanolamine-phosphotransferase, the gene accession number was listed in the Supplementary Table S2.

To understand the mechanism of glycerophospholipids accumulation better, transcriptome data from first-, second-, and third-instar larvae were analyzed to evaluate the expression levels of genes involved in glycerophospholipid biosynthesis (Figure 3). The key glycerophospholipid synthases, such as glycerol kinase (EG-A), glycerol-3-phosphate O-acyltransferase (EG-B), phosphatidate phosphatase (EG-C), phosphatidate cytidylyltransferase (EG-D), glycerol-3-phosphate 3-phosphatidytransferase (EG-E), and ethanolamine phosphotransferase (EG-F), were identified in the pathway (Figure 3A). The expression levels of genes that encoded all glycerol kinases were up-regulated significantly with increasing instar stages (Figure 3B). The genes that encoded phosphatidate cytidylyltransferase and ethanolamine-phosphotransferase had similar expression patterns and showed the highest expression levels in the third-instar larvae. In addition, some glycerol-3-phosphate O-acyltransferase genes and phosphatidate phosphatase genes as well as one glycerol-3-phosphate 3-phosphatidytransferase gene showed an up-regulated expression pattern with increasing instar stages. Most of the glycerol-3-phosphate O-acyltransferase genes and phosphatidate phosphatase genes exhibited no significant differences among different instars.

## 4. Discussion

*A. dichotoma* has attracted the attention of researchers because of its potential application in the development of food and medicines [14]. Extracts from *A. dichotoma* larvae have been shown to exhibit hepatoprotective, anticancer, antidementia, antiobesity, and antioxidant activity [14,16,23]. Thus, it is desirable to investigate the bioactive constituents of *A. dichotoma* larvae. For example, in [18], inosine was isolated from larvae of *A. dichotoma*, 32 different volatile oils were identified, and the fatty acid profile of A. dichotoma larvae was also identified. In this study, eight glycerophospholipid classes that contained a total of 833 species were identified in *A. dichotoma* larvae, far more than the quantity (only 10) of glycerophospholipids identified in *Eupolyphaga sinensis* [24]. The presence of nutritional and functional constituents supports the idea that these larvae could serve as a valuable resource for food and/or industrial purposes.

The glycerophospholipid fractions of *A. dichotoma* larvae in all stages were represented mainly by PE, followed by PC and PG. This result did not align with the findings previously reported for crickets, *Drosophila melanogaster*, and *Hermetia illucens* larvae, in which PC and PE were the primary classes and made up the largest portion of glycerophospholipids [4,6,25]. Similarly, PC was also the predominant lipid class in honeybee (*Apis mellifera*) workers [26].

Together with PC, PE is found in all living organisms, and it represents the backbone of most biological membranes [27,28,29]. PE serves as a supplement to aid in promoting healthy brain function, enhancing cognitive performance, and decreasing inflammation. It is also beneficial for liver health, lowers cholesterol, and supports cardiovascular health [30]. PC, which is also known as lecithin, serves as a surfactant in food, providing antioxidant properties, flavor protection, and emulsification in a wide range of food items [4].

Choline, vital for many physiological activities, primarily comes from dietary PC [31,32]. The neurotransmitter acetylcholine is derived from choline. Moreover, choline deficiency may impair the metabolism of hepatic lipids. Humans can synthesize choline endogenously; however, the amount produced is insufficient to satisfy daily needs, especially in pregnant and lactating women [33]. Currently, lecithin is extracted mainly from crude soybean oil. This method has many disadvantages, including limited traceability, the use of genetically modified soybeans, and the increasingly frequent instances of allergic reactions to soybeans [4,34]. Therefore, the utilization of insects is now seen as a promising alternative source of lecithin.

To assess applications of lecithin from *A. dichotoma* larvae in foods, pharmaceuticals, or cosmetics, further studies are necessary to better understand its extraction and its functional properties. In addition, PI serves important physiological functions and has unique emulsifying properties; it can be used in many fields such as food, nutraceuticals, and pharmaceuticals [35]. *A. dichotoma* larvae have the potential to be a beneficial food product.

Lipid metabolism and transformation are vital to insects. Feeding on resistant rice, the brown planthopper (*Nilaparvata lugens* Stål) accelerated triglyceride (TG) mobilization to supply energy for growth, development, and egg production [36]. During the development of *H. illucens* larvae, the majority of GP classes, particularly PC and PE, showed a decline [6]. During the growth process of adult fruit flies, levels of GPs like PC and PE decline [25]. Also, in pork, the amount of glycerophospholipids decreased with an increase in age [6]. During egg yolk formation, the relative abundance of PC decreased, whereas that of PE increased [37]. Contrarily, the total content of GPs in *A. dichotoma* larvae was higher significantly at the third instar stage; moreover, the content of most GP classes, especially PC and PE, increased in the third instar stage, compared with first- and second-instar larvae. PCA analysis indicated that the glycerophospholipid profile was clearly distinguishable across sample groups (F = 57.506, *p* < 0.001). These results indicated that the glycerophospholipid profile was rearranged with development of *A. dichotoma* larvae. Even though the physiological and biological functions of GP transformation during the growth of *A. dichotoma* larvae are still unknown, the composition of GPs of *A. dichotoma* larvae at different instars offers valuable insights for utilizing this insect.

Genes that encoded glycerol kinase, glycerol-3-phosphate O-acyltransferase, and 1-acyl-sn-glycerol-3-phosphate acyltransferase showed significantly up-regulated expression (Figure 2). These enzymes contribute to the synthesis of PA from glycerol [38]. PA is the smallest and simplest glycerophospholipid, and so it is the precursor for other more complex glycerophospholipids, which include PC and PE. However, the content of PA did not increase very much even though it showed an increased pattern.

We speculate that PA was converted to PC or PE by the CDP-DG pathway. This hypothesis is also supported by the expression of genes that encoded phosphatidate phosphatase, phosphatidate cytidylyltransferase, and ethanolamine-phosphotransferase, which were up-regulated significantly and were involved in the biosynthesis of PC and PE [39,40]. Other glycerophospholipids like PI and PS exhibited a very similar pattern with PA. Conversely, PG and PIP were down-regulated significantly with development of *A. dichotoma* larvae. However, the precise function of the glycerophospholipid synthetases need to be validated further to determine the mechanism of transformation of glycerophospholipids.

## 5. Conclusions

In this study, we conducted the initial global lipidome for *A. dichotoma* larvae. We identified glycerophospholipids directly, and tracked their variations during three developmental stages of *A. dichotoma* using a lipidomic technique. Interestingly, large amounts of PC and PE accumulated in *A. dichotoma* larvae, especially in third-instar larvae. Thus, lipidomics and transcriptomics provide a practical approach to study *A. dichotoma* larvae and to explore the molecular processes of glycerophospholipid accumulation as they grow. The results offer new perspectives on the possible nutritional benefits and full utilization of *A. dichotoma* larvae.

## Figures and Tables

**Table 1:** Glycerophospholipid profiles of *Allomyrina dichotoma* larvae.

Class	Number	Content (ug/g)	Percent (%)
CL	72	4384.64	2.83
PA	14	549.39	0.35
PC	328	51,155.36	33.03
PE	223	73,696.16	47.58
PG	84	15,125.26	9.77
PI	57	3802.01	2.45
PIP	21	2899.63	1.87
PS	34	3267.48	2.11
Total	833	15,4879.93	100.00

## Data Availability

The original contributions presented in this study are included in the article. Further inquiries can be directed to the corresponding author.

## Supplementary Materials

The following supporting information can be downloaded at: https://www.mdpi.com/article/10.3390/insects16121220/s1, Figure S1: Base Peak Chromatograms (BPC) for QC samples in positive ion modes (POS) and negative ion modes (NEG); Figure S2: Correlation plot for QC samples; Figure S3: Multivariate Control Chart for QC samples; Figure S4: Relative standard deviation (RSD) curve for QC samples; Figure S5: Principal component analysis for all samples; Figure S6: Hotelling-s T2 Range Line Plot for all samples. Table S1: Internal standard mapping each lipid class; Table S2: Accession number for genes in the heatmaps.
