# Supplementary material for: Glycerophospholipid Profiles of Allomyrina dichotoma Larvae at Different Instars Based on Lipidomics and Transcriptomics Suggest a Promising Lipid Source
Source: Insects. 2025 Nov 29;16(12):1220. doi: 10.3390/insects16121220 (PMC12733726; doi:10.3390/insects16121220)
Supplement: Supplementary file 1 [file insects-16-01220-s001.zip › Supplementary Figure S1-S6.pdf]

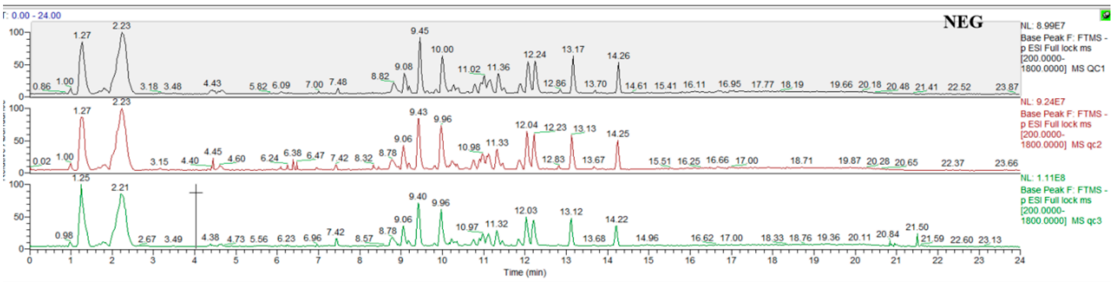

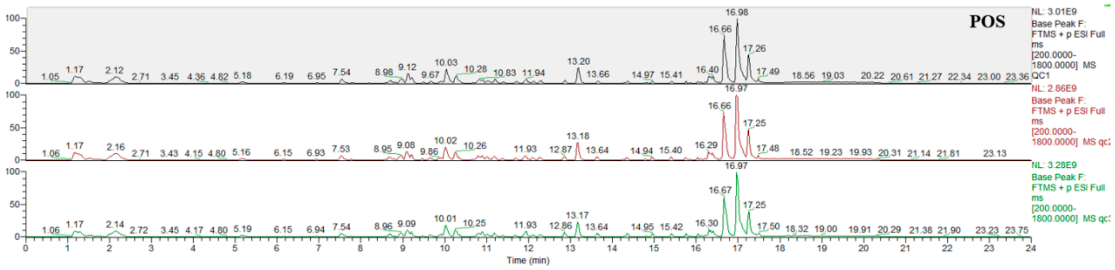

Supplementary Figure S1. Base Peak Chromatograms (BPC) for QC samples in positive ion modes (POS) and negative ion modes (NEG).

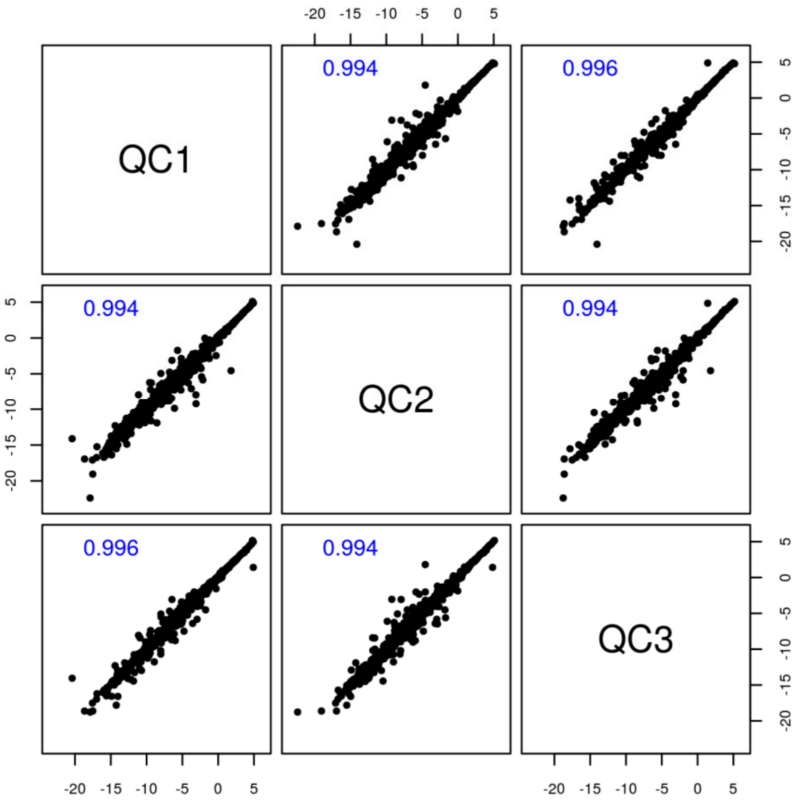

Supplementary Figure S2. Correlation plot for QC samples.

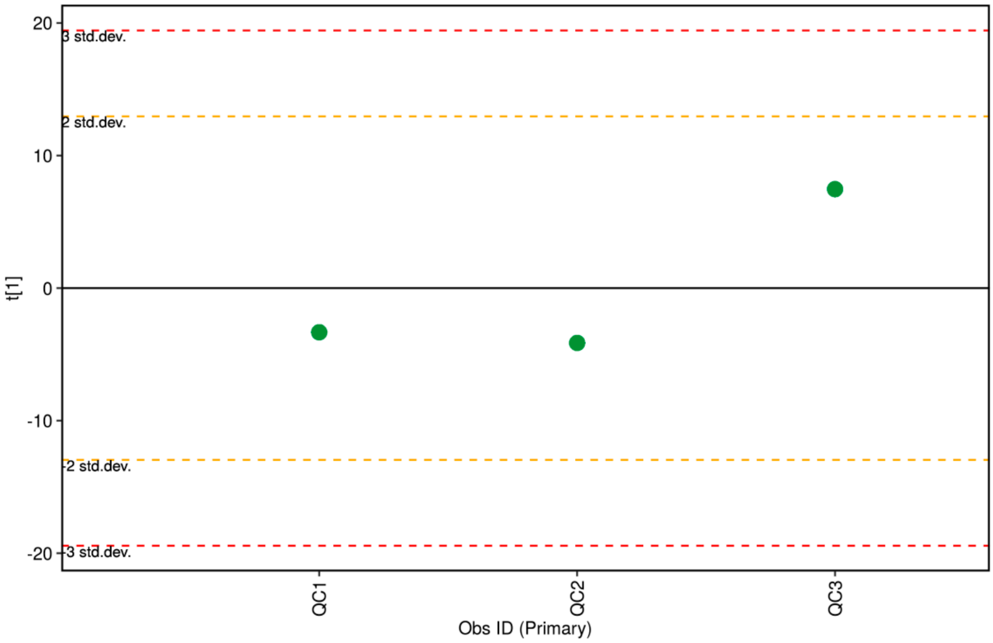

Supplementary Figure S3. Multivariate Control Chart for QC samples.

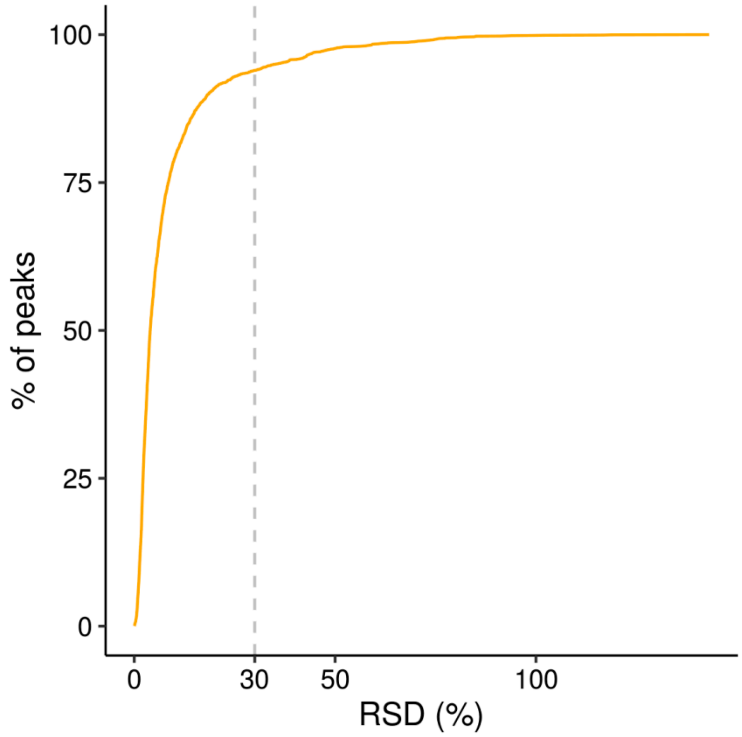

Supplementary Figure S4. Relative standard deviation (RSD) curve for QC samples.

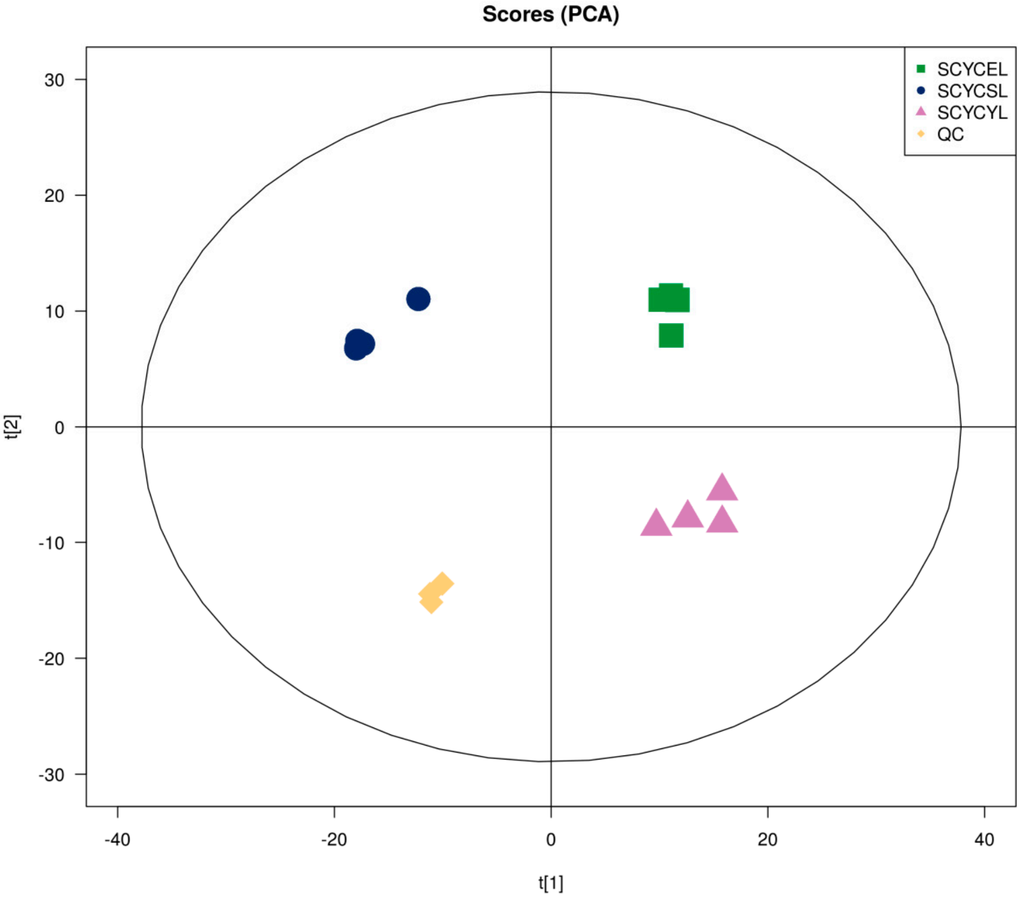

Supplementary Figure S5. Principal component analysis for all samples.

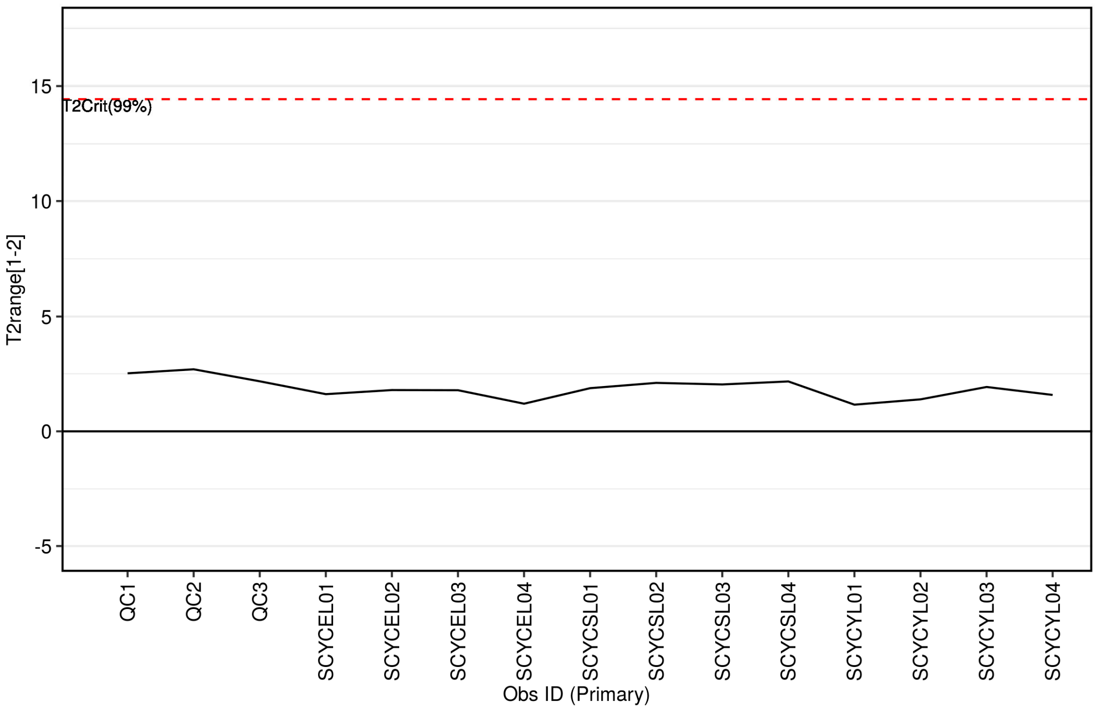

Supplementary Figure S6. Hotelling-s T2 Range Line Plot for all samples.
