# Supplementary material for: Glycerophospholipid Profiles of Allomyrina dichotoma Larvae at Different Instars Based on Lipidomics and Transcriptomics Suggest a Promising Lipid Source
Source: Insects. 2025 Nov 29;16(12):1220. doi: 10.3390/insects16121220 (PMC12733726; doi:10.3390/insects16121220)
Supplement: Supplementary file 1 [file insects-16-01220-s001.zip › Supplementary Table S1. Internal standard mapping each lipid class..pdf]

Supplementary Table S1. Internal standard mapping each lipid class.

**Class**	**Internal Standard**
CL	15:0-18:1(d7)PE
PA	15:0-18:1(d7)PA
PC	15:0-18:1(d7)PC
PE	15:0-18:1(d7)PE
PG	15:0-18:1(d7)PG
PI	15:0-18:1(d7)PI
PIP	15:0-18:1(d7)PI
PS	15:0-18:1(d7)PS
