# Supplementary material for: Glycerophospholipid Profiles of Allomyrina dichotoma Larvae at Different Instars Based on Lipidomics and Transcriptomics Suggest a Promising Lipid Source
Source: Insects. 2025 Nov 29;16(12):1220. doi: 10.3390/insects16121220 (PMC12733726; doi:10.3390/insects16121220)
Supplement: Supplementary file 1 [file insects-16-01220-s001.zip › Supplementary Table S2.pdf]

Supplementary Table S2. Accession number for genes in the heatmaps.

Transcriptomic ID	Accession number	Annotation
TRINITY_DN1671_c0_g1	SAMN53308973	glycerol kinase
TRINITY_DN7263_c0_g1	SAMN53308974	glycerol kinase
TRINITY_DN379_c1_g1	SAMN53308975	glycerol kinase
TRINITY_DN3795_c0_g1	SAMN53308976	glycerol kinase
TRINITY_DN9048_c0_g1	SAMN53308977	glycerol-3-phosphate O-acyltransferase 1/2
TRINITY_DN439_c0_g2	SAMN53308978	glycerol-3-phosphate O-acyltransferase 1/2
TRINITY_DN7256_c0_g1	SAMN53308979	glycerol-3-phosphate O-acyltransferase 1/2
TRINITY_DN438_c0_g1	SAMN53308980	glycerol-3-phosphate O-acyltransferase 1/2
TRINITY_DN3257_c0_g1	SAMN53308981	glycerol-3-phosphate O-acyltransferase 1/2
TRINITY_DN36967_c0_g1	SAMN53308982	glycerol-3-phosphate O-acyltransferase 1/2
TRINITY_DN11600_c0_g1	SAMN53308983	glycerol-3-phosphate O-acyltransferase 1/2
TRINITY_DN10064_c0_g1	SAMN53308984	phosphatidate phosphatase
TRINITY_DN75_c0_g2	SAMN53308985	phosphatidate phosphatase
TRINITY_DN50522_c0_g1	SAMN53308986	phosphatidate phosphatase
TRINITY_DN2667_c0_g3	SAMN53308987	phosphatidate phosphatase
TRINITY_DN47935_c0_g1	SAMN53308988	phosphatidate phosphatase
TRINITY_DN426_c5_g1	SAMN53308989	phosphatidate-cytidylyltransferase
TRINITY_DN12551_c0_g1	SAMN53308990	glycerol-3-phosphate 3-phosphatidyltransferase
TRINITY_DN19341_c0_g1	SAMN53308991	glycerol-3-phosphate 3-phosphatidyltransferase
TRINITY_DN52019_c0_g1	SAMN53308992	ethanolamine-phosphotransferase
